# Comparison of platforms for testing antibodies to *Chlamydia trachomatis* antigens in the Democratic Republic of the Congo and Togo

**DOI:** 10.1038/s41598-021-86639-8

**Published:** 2021-03-31

**Authors:** Sarah Gwyn, Marcel S. Awoussi, Ana Bakhtiari, Rachel N. Bronzan, Kathryn Crowley, Emma M. Harding-Esch, Yao Kassankogno, Janvier N. Kilangalanga, Felix Makangila, Sylvain Mupoyi, Jeremiah Ngondi, Bonaventure Ngoyi, Stephanie Palmer, Jessica M. Randall, Anders Seim, Anthony W. Solomon, Raymond Stewart, Kwamy Togbey, Pitchouna A. Uvon, Diana L. Martin

**Affiliations:** 1 Division of Parasitic Diseases and Malaria, Centers for Disease Control and Prevention, 1600 Clifton Rd NE B23 Room 10-113, Atlanta, GA 30029 USA; 2 Ministère de la Santé et de L’Hygiène Publique, Lomé, Togo; 3 Task Force for Global Health, Decatur, GA USA; 4 Health and Development International, Newburyport, MA USA; 5 RTI International, Washington, DC USA; 6 Clinical Research Department, London School of Hygiene & Tropical Medicine, London, UK; 7 Health and Development International, Lome, Togo; 8 Saint Joseph Hospital, Kinshasa, Democratic Republic of the Congo; 9 Ministry of Public Health, Kinshasa, Democratic Republic of the Congo; 10 RTI International, Kinshasa, Democratic Republic of the Congo; 11 FHI360, Washington, DC USA; 12 Emory University, Atlanta, GA USA; 13 Department of Control of Neglected Tropical Diseases, World Health Organization, Geneva, Switzerland; 14 Programme National des Maladies Tropicales Négligées, Ministère de la Santé et de l’Hygiène Publique, Lomé, Togo

**Keywords:** Bacterial infection, Public health, Antibodies

## Abstract

Trachoma, caused by repeated ocular infection with *Chlamydia trachomatis* (Ct), is targeted for elimination as a public health problem. Serological testing for antibodies is promising for surveillance; determining useful thresholds will require collection of serological data from settings with different prevalence of the indicator trachomatous inflammation—follicular (TF). Dried blood spots were collected during trachoma mapping in two districts each of Togo and Democratic Republic of the Congo. Anti-Ct antibodies were detected by multiplex bead assay (MBA) and three different lateral flow assays (LFA) and seroprevalence and seroconversion rate (SCR) were determined. By most tests, the district with > 5% TF (the elimination threshold) had five–sixfold higher seroprevalence and tenfold higher SCR than districts with < 5% TF. The agreement between LFA and MBA was improved using a black latex developing reagent. These data show optimization of antibody tests against Ct to better differentiate districts above or below trachoma elimination thresholds.

## Introduction

Trachoma, an eye disease caused by repeated ocular infection with the bacterium *Chlamydia trachomatis (Ct)*, is the leading infectious cause of blindness and is targeted for elimination as a public health problem. The elimination target of less than 5% trachomatous inflammation—follicular (TF) in children aged 1–9 years leaves open the possibility of low-level transmission after the cessation of interventions. These interventions—mass drug administration (MDA) of antibiotics and campaigns to encourage facial cleanliness and environmental improvement—comprise the A, F, and E components of the SAFE strategy (“S” is for surgery to correct morbidity associated with in-turned eyelashes that rub against the eyeball: trichiasis)^1^. Tools are needed to monitor for recrudescence in settings where elimination of trachoma as a public health problem has been validated.

A growing body of evidence demonstrating the utility of serology for post-validation surveillance is being generated^2–9^. While serology has no diagnostic utility for trachoma at an individual level, the longevity of the anti-*Ct* antibody responses is appropriate for assessing trends in transmission at population level over time. This may be particularly apposite for trachoma, for which host immune responses to repeated ocular *Ct* infections are critical for development of pathology^10–13^. One important question is an appropriate threshold of seropositivity to trigger-decision-making by trachoma programs. Preliminary models from multi-country serological data analyses suggest that a mean seroprevalence less than 6.2% and a seroconversion rate (SCR) of below 1.5 per 100 individuals per year in 1–9-year-olds correspond to TF < 5%^6^. However, more data are needed to evaluate the relationship between overall seroprevalence, SCR and TF prevalence in settings at various stages of the elimination process.

Another important consideration for serosurveillance studies is the choice of test to measure anti-*Ct* antibodies. Because of the discordance between the longevity of infection and antibody positivity, there is no gold standard for antibody tests—i.e., a test that can tell who has been exposed sufficiently to generate an antibody response. Antibodies against Pgp3 were first identified by ELISA^14^, and subsequently have undergone various revisions^15–17^. We previously adapted testing for anti-Pgp3 antibodies to a multiplex bead assay (MBA), in order to allow evaluation of antibody tests in trachoma-endemic settings within a multiplexed, integrated, serological surveillance platform^3^. This was later modified to an ELISA with a series of plate control standards to normalize absorbance values and thereby standardize testing between laboratories^15^. The test was also adapted to a lateral flow assay (LFA) to provide a rapid, low-cost, low-technical capacity alternative to ELISAs or MBA^18,19^.

As tests are trialed in an increasing variety of epidemiological settings, it is important to use the data and user feedback to undertake further test optimization, and ultimately work towards rationalizing the menu of options available while consensus emerges on the target product profile. Here, we compare seroconversion rate (SCR) and seroprevalence estimates from four evaluation units in two countries undergoing baseline mapping for trachoma (Togo and Democratic Republic of the Congo [DRC]) using multiple versions of the LFA and the MBA, including an improved version of the LFA that employs black latex as the developing reagent.

## Methods

### Ethics

Ethical approval for individual studies was given by institutional review boards at the Togo Ministry of Health and Social Protection and the Ethics Committee of the Ministry of Public Health of the Democratic Republic of the Congo. Written informed consent from parents was obtained for study participants, all of whom were aged < 18 years. Tropical Data has ethics approval from the London School of Hygiene & Tropical Medicine to support health ministries to conduct trachoma prevalence surveys. CDC staff did not interact with study participants or have access to identifying information and were considered to be non-engaged in research. All methods were carried out in accordance with relevant guidelines and regulations.

### Study sites

In general, an evaluation unit (EU) is a district. For trachoma elimination purposes, WHO defines a district as the normal “administrative unit for health care management [which] for purposes of clarification consists of a population between 100,000–250,000 persons”^20^. In Togo, baseline mapping was conducted in seven districts in August–September 2017 to determine possible needs for intervention; in 2 of those districts, fingerprick blood was collected to create dried blood spots (DBS) and conduct field testing of the Pgp3 lateral flow assay. In DRC, DBS were collected in June 2018 as part of baseline mapping in 2 health zones of Tanganyika Province. The underlying surveys were conducted in accordance with WHO recommendations for trachoma prevalence surveys^21^ with the research element built onto that scaffold. In each EU, a two-stage cluster random sampling design was used to sample 25 villages (clusters) with probability of selection proportional to population size, and 30 households per cluster. In one EU (Nyemba) only 24 clusters were included in the analysis as one cluster was inadvertently surveyed twice. All household residents aged 1 year or more were examined for clinical signs of trachoma.

### TF grading

Graders for all studies underwent training from certified Tropical Data trainers, using international protocols developed by the Global Trachoma Mapping Project^22^. TF was defined as the presence of 5 or more follicles, each at least 0.5 mm in diameter, in the central part of the upper tarsal conjunctiva of one or both eyes^22^. Data were entered into standardized forms on Android smartphones by Tropical Data-certified data recorders. Data recording, cleaning and analysis were done according to published quality assurance mechanisms^23^.

### DBS collection

Fingerprick blood was collected onto filter paper containing 6 circular extensions calibrated to each absorb 10 µL of blood (TropBio Pty Ltd., Townsville, Queensland, Australia). In DRC, filter paper wheels were dried overnight, placed in sealed plastic bags with desiccant and stored in coolers with vaccine cold accumulators for transport from the field to the provincial general reference hospital where the DBS were placed in cold storage at − 20 °C. The DBS were transported from the provincial hospital to the Department of Tropical Medicine of the University of Kinshasa and put into cold storage and DBS were stored long term at − 20 °C with desiccant. DBS were shipped at ambient temperatures to CDC for analysis. In Togo, DBS were dried in the field at ambient temperature and then overnight at 2–8 °C. DBS were then placed in sealed plastic bags with desiccant and kept refrigerated. At the end of the survey, samples were sent to the INH (National Institute of Hygiene) in Lomé and stored at − 20 °C. All DBS specimens were shipped at ambient temperatures to CDC with desiccants and moisture indicators for analysis.

### MBA

Each DBS extension was eluted overnight at 4 °C in PBS containing 0.5% casein, 0.3% Tween 20, 0.5% polyvinyl alcohol, 0.8% polyvinylpyrrolidone, 0.02% sodium azide, and 3 µg/mL *E. coli* extract (Buffer B). DBS eluates were diluted in Buffer B to a final dilution of 1:400. Dilutions were tested on the multiplex bead assay as previously described^3^. Pgp3 and CT694-coupled beads (1250 per antigen per well) were incubated for 1.5 h with 50 µL of diluted sample. Wells were washed 3 times with PBST (0.3% Tween 20) and incubated with 50 ng biotinylated mouse anti-human IgG (Southern BioTech, Birmingham, AL) and 20 ng biotinylated mouse anti-human IgG4 (Southern BioTech) for 45 min to detect any Pgp3 and CT694 specific IgG bound to the beads. After 3 washes with PBST, wells were incubated with 250 ng phycoerythrin-labeled streptavidin (Invitrogen, South San Francisco, CA) for 30 min. Wells were washed 3 times with PBST and incubated with 50 µL PBS containing 0.5% BSA, 0.05% Tween-20 and 0.02% sodium azide to remove any loosely bound antibodies. After one more wash with PBST, wells were suspended in 100 µL PBS and plates were stored overnight at 4 °C. The next day, plates were read on a Bio-Plex 200 instrument (Bio-Rad, Hercules, CA) equipped with Bio-Plex manager 6.0 software (Bio-Rad). The median fluorescence intensity (MFI) with the background from the blank well (Buffer B alone) subtracted out (MFI-bg) was recorded for each antigen for each sample. The cutoff of positivity was established as an MFI-bg of 1647 for Pgp3 and 347 for CT694 by using receiver operating characteristic curve analysis on a panel of 101 samples from ocular *Ct* PCR-positive individuals from the United Republic of Tanzania and 74 pediatric samples from New York, NY USA which tested negative by a chlamydial microimmunofluorescence assay.

### LFA

#### Manufacturing

Pgp3 LFAs in cassettes for field testing in Togo were manufactured at CDC as previously described^18^. The production of Pgp3 LFA-dipsticks at CDC for the LFA-gold assay has also been previously described^19^. Pgp3 LFA-dipsticks for the LFA-latex assay were manufactured at CDC as follows: a nitrocellulose membrane (Sartorius, Bohemia, NY) and an absorbent pad were placed on a backing card with a 1–2 mm overlap. Pgp3 protein (0.5 mg/mL) and biotinylated bovine serum albumin (BSA-biotin, 1.0 mg/mL; Arista Biologicals, Allentown, PA) were dispensed onto the nitrocellulose membrane at a rate of 0.1 µL/mm using a BioDot XYZ 3060 dispenser (BioDot, Irvine, CA) and then dried overnight in a desiccator cabinet with a relative humidity of less than 20%. Dried membranes were cut into 4 mm strips using an A-point guillotine cutter (Arista Biologicals) and stored at room temperature in a desiccator cabinet with a relative humidity of less than 20%.

#### Training

All field and laboratory LFAs were read by staff who had achieved a score of κ > 0.80 on a panel of 30 dried blood spot specimens compared to the trainer.

#### LFA-field

Blood was run on the Pgp3 LFA cassette in Togo as previously described^18^. Fingerprick blood (20 µL) was transferred into the sample port of the Pgp3 LFA cassette using a micropipette (Safe-Tec, Ivyland, PA). Chase buffer (0.3% Tween 20 in PBS) was added to the buffer port and tests were read as positive, negative or invalid 30 min later. Due to time constraints in constructing LFA cassettes, this test was run only on the first 1000 participants in each district.

#### LFA-gold

DBS were tested by the LFA-dipstick assay using a gold detector reagent^19^. Each DBS extension was eluted in 60 µL of LFA buffer (0.5% BSA, 0.3–1% Tween-20, 0.02% sodium azide in PBS) for 4–24 h in a flat-bottom 96-well plate (USA Scientific, Ocala, FL). Streptavidin (SA)-gold conjugate (Arista Biologicals) and Pgp3-gold conjugate (Expedeon, San Diego, CA) were added to each well no more than 4 h prior to testing. Pgp3 LFA-dipsticks were placed into each well for 15–20 min. Then, 40 µL of LFA buffer was added. After 5–10 min, LFAs were removed and read as positive, negative or invalid.

#### LFA-latex

DBS were tested by the LFA-dipstick assay using a black latex detector reagent. Pgp3-latex (Expedeon) and SA-gold (Arista Biologicals) were diluted 1:240 and 1:120, respectively, in PBST (0.3% Tween-20 in PBS) to create a conjugate mastermix. Each DBS was eluted in 60 µL of conjugate mastermix in a well of a flat-bottom 96-well plate, overnight at 4 °C. Pgp3 LFA-dipsticks were added to each well and incubated for 15 min, until all the liquid was absorbed. PBST (80 µL) was then added to each well to clear the background caused by hemolyzed red blood cells on the nitrocellulose membrane. Each LFA was read as positive, negative or invalid once the background was completely cleared, which took about 5 min.

### Statistical analysis

TF prevalence estimates were calculated using standard Tropical Data analyses, using the mean of the age-standardized cluster-level TF proportions (https://github.com/itidat/tropical-data-analysis-public). 95% confidence intervals (CIs) were calculated by taking the 2.5th and 97.5th centiles of all ordered results after bootstrapping the cluster means over 10,000 iterations. Overall seroprevalence estimates for 1–9-year-olds were calculated using age-standardized district-level seroprevalence proportions. Seroconversion rates were calculated using R (version 3.6.3), as previously described (https://github.com/jessmrandall/CDC_Multicountry_trachoma_seroproject)^6^.

### Conference presentation

Portions of the data were presented at the Programme Managers Meeting to Accelerate Control and Elimination of Neglected Tropical Diseases in the Western Pacific Region, held virtually September 1–4, 2020.

## Results

### Demographic information and TF data

#### Togo

**Figure 1:**
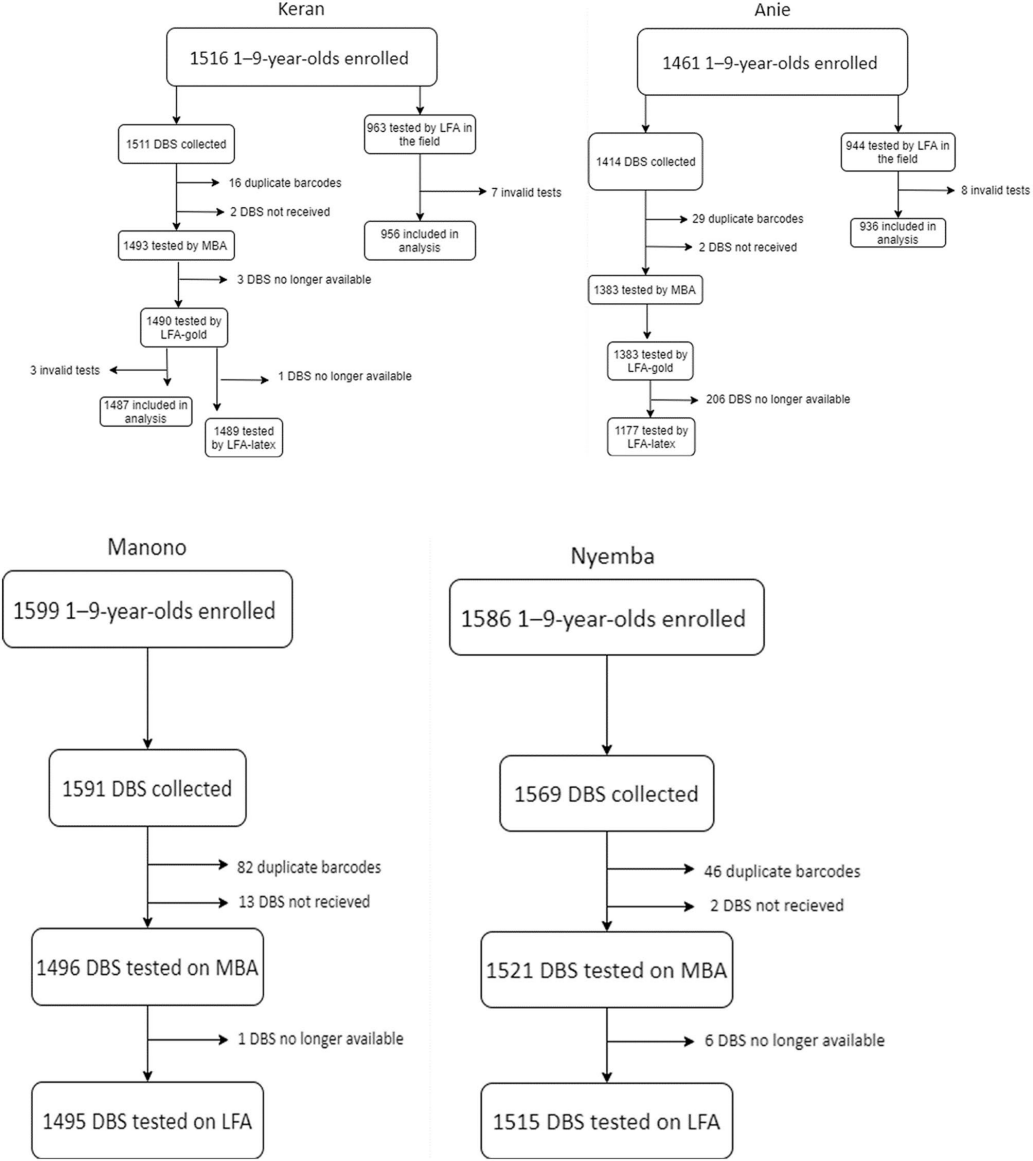
Flow chart of sample collection in each evaluation unit. Figure was created using draw.io version 14.3.1 (https://app.diagrams.net/).

**Figure 2:**
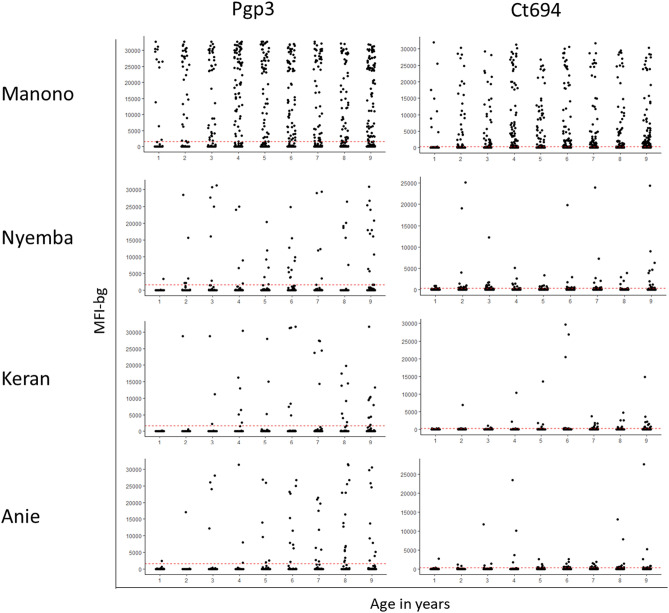
Intensity of antibody response (determined in the multiplex bead assay [MBA]) by age in each participating evaluation unit. Manono health zone, Democratic Republic of the Congo (DRC); Nyemba health zone, DRC; Keran district, Togo; Anie district, Togo. In each panel, the y-axis shows median fluorescence intensity with background subtracted (MFI-bg), and the x-axis shows age in years. Graphs were created using R version 3.6.3 (https://www.r-project.org/).

**Figure 3:**
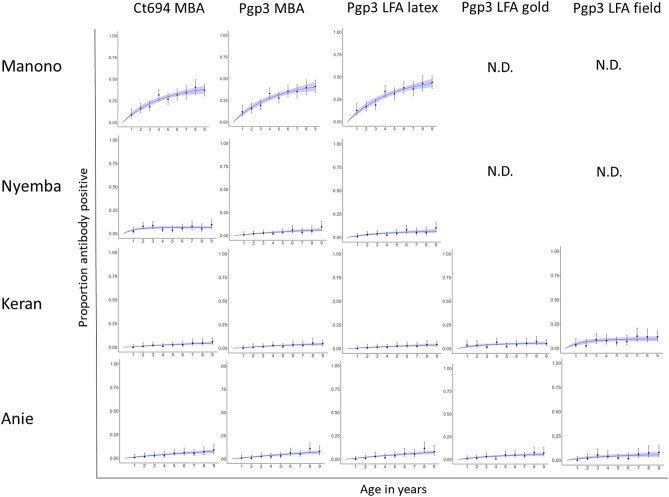
Proportion antibody positive by age in each participating evaluation unit. Manono health zone, Democratic Republic of the Congo (DRC); Nyemba health zone, DRC; Keran district, Togo; Anie district, Togo. *MBA* multiplex bead assay, *LFA* lateral flow assay, *N.D.* not done. Graphs were created using R version 3.6.3 (https://www.r-project.org/).

In Keran district, a total of 1516 1–9-year-olds were enrolled in the study. Of these individuals, 788 (52.0%) were male, and 1511 (99.7%) were examined and had DBS collected. In Anie district a total of 1461 1–9-year-olds were enrolled in the study. Of these individuals, 708 (48.5%) were male, and 1414 (96.8%) were examined and had DBS collected. The number of samples tested by each assay in each district is shown in Fig. 1. The age-standardized prevalence of TF in 1–9-year-olds was 0.4% [95% CI 0.1–0.6%] in Keran and 0.3% [95% CI 0.1–0.6%] in Anie (Table 1). Intensity of antibody responses by year of age are shown in Fig. 2, and the proportion antibody positive by year of age is shown in Fig. 3.

**Table 1:** Age-adjusted prevalence of trachomatous inflammation—follicular (TF) and anti-Ct antibodies by different immunoassays.

Country	EU	TF% (95% CI)	CT694 MBA% (95% CI)	Pgp3 MBA% (95% CI)	Pgp3 LFA cassette% (95% CI)	Pgp3 LFA gold% (95% CI)	Pgp3 LFA black latex% (95% CI)
Togo	Keran	0.4 (0.1–0.6)	2.6 (1.2–6.3)	2.8 (1.3–6.5)	8.1 (4.4–14.9)	4.6 (2.3–8.9)	2.4 (1.0–6.0)
	Anie	0.3 (0·1–0·6)	4.5 (2.3–9.0)	4.5 (2.4–8.9)	4.2 (1.9–9.8)	4.0 (2.0–8.2)	4.5 (2.3–9.4)
DRC	Manono	7.3 (4.2–11.6)	26.9 (20.9–34.0)	28.3 (22.2–35.5)	ND	ND	30.0 (23.7–37.2)
	Nyemba	1.1 (0.5–1.8)	6.0 (3.4–10.7)	3.9 (1.9–8.0)	ND	ND	4.7 (2.5–9.0)

*EU* evaluation unit, *CI* confidence interval, *Pgp3* plasmid gene product 3, *Ct* *Chlamydia trachomatis,* *MBA* multiplex bead assay, *LFA* lateral flow assay, *ND* not done.

#### DRC

In Manono, a total of 1599 1–9-year-olds were enrolled in the study. Of these individuals, 784 (49%) were male, 1595 (99.7%) were examined and 1591 (99%) had DBS collected. In Nyemba, a total of 1586 1–9-year-olds were enrolled in the study. Of these individuals, 846 (53.3%) were male, 1578 (99.5%) were examined, and 1569 (98.9%) had DBS collected. The number of samples tested by each assay in each district is shown in Fig. 1. The prevalence of 1–9-year-olds with TF was 7.3% [95% CI 4.2–12.0%] in Manono and 1.1% [95% CI 0.5–1.8%] in Nyemba (Table 1). Intensity of antibody responses by year of age are shown in Fig. 2, and the proportion antibody positive by year of age is shown in Fig. 3.

### Seroprevalence estimates

Table 1 shows the age-adjusted seroprevalence in 1–9-year-olds by test format for each EU included. The EU with TF > 5% (Manono) had a seroprevalence of > 25% on each of the assays (Table 1). The EUs with TF < 5% had a seroprevalence < 5% on all assays except using the LFA cassette in Keran (8.1%, 95% CI 4.4–14.9%) and CT694 MBA in Nyemba (6.0%, 95% CI 3.4–10.7%). Confidence intervals were overlapping between Pgp3 MBA and each type of test for each EU.

### Seroconversion rate estimates

Table 2 shows the seroconversion rate per 100 children per year for each test format in each EU. The EU with TF > 5% (Manono) had seroconversion rates above 10 on all tests. The EUs with TF < 5% had seroconversion rates below 2 for Pgp3 MBA and Pgp3 LFA latex but not CT694 MBA (Nyemba), Pgp3 LFA cassette (Keran) or Pgp3 LFA gold (Keran) (Table 2).

**Table 2:** Seroconversion rates (SCR) by different immunoassays.

Country	EU	CT694 MBA (95% CI)	Pgp3 MBA (95% CI)	Pgp3 LFA cassette (95% CI)	Pgp3 LFA gold (95% CI)	Pgp3 LFA latex (95% CI)
Togo	Keran	0.7 (0.5–1.0)	0.7 (0.5–1.2)	5.8 (3.0–10.2)	2.3 (1.2–3.9)	0.6 (0.4–1.2)
	Anie	1.1 (0.8–2.0)	1.0 (0.8–1.7)	1.9 (0.8–3.3)	1.3 (0.7–2.8)	1.1 (0.8–1.7)
DRC	Manono	10.1 (8.1–14.1)	10.4 (8.0–13.8)	ND	ND	10.8 (8.4–14.6)
	Nyemba	6.2 (4.2–8.4)	1.1 (0.8–1.6)	ND	ND	1.8 (1.1–3.2)

*EU* evaluation unit, *CI* confidence interval, *Pgp3* plasmid gene product 3, *CT* *Chlamydia trachomatis,* *MBA* multiplex bead assay, *LFA* lateral flow assay, *ND* not done.

## Discussion

Serology is likely to be an important component of surveillance in settings that have eliminated trachoma as a public health problem. It is also likely to prove useful in countries in which some EUs have met the TF elimination prevalence target, but others have not. Serosurveillance has the advantage of using a sample type (blood or blood products) that is often collected for other disease surveillance; trachoma programs could therefore potentially use stored specimens, saving resources. But for serology to be widely adapted for trachoma surveillance, we must first demonstrate that serological tests are fit for purpose by evaluating their use in population-based surveys in trachoma-endemic and non-endemic areas. Here we show distinct differences in seroprevalence and SCR in an EU with a relatively low TF prevalence (Manono, DRC, at 7.3% TF) that falls above the elimination threshold compared to three other districts with TF < 5%. These data contribute to the growing body of evidence that serological data (expressed as either a seroprevalence or seroconversion rate, and generated by either rapid or bead-based tests) reflect population-level TF prevalence, and probably provide meaningful signals about transmission intensity of ocular *Ct*. An empirical comparison of serological data with the parameter likely to be of pathological significance—the incidence of ocular *Ct* infection, determined at population level—would be exceedingly difficult to undertake.

Seroprevalence in the three districts with TF < 5% in this study was similar to that observed in pre-validation surveys in Ghana^24^, Nepal^8^, and the United Republic of Tanzania^7^. The data from Manono, where TF prevalence was 7.3%, were similar to those from 1–5-year-olds in Niger when TF prevalence was 7.5% after 3 rounds of antibiotic MDA^25^. For the Pgp3 LFA cassette in Keran with TF < 5%, the seroprevalence and SCR were both higher than the predicted levels for countries in which trachoma is no longer a public health problem (6.2% seroprevalence; SCR 1.5^6^). These early modeling predictions will be improved by the inclusion of serological data from countries from sub-Saharan Africa, which carries the largest trachoma burden^26^. Existing models are over-informed by data from Pacific Island nations, which were early adopters of serological testing as a means to understand the local discordance between high TF prevalences in children and low prevalences of trachomatous trichiasis and trachoma-related blindness in adults^27–29^. The addition of serology testing to baseline surveys in pre-intervention trachoma-endemic populations has been identified as an operational research priority^9^. The rapid implementation of baseline trachoma mapping within the Global Trachoma Mapping Project from 2012–2016^30^ now leaves few suspected-endemic areas remaining to be mapped, so the current data are likely to be especially valuable for informing appropriate thresholds for the use of trachoma serology.

There were several limitations to the current work. The Togo data are limited by having fewer LFA tests run in the field than in the laboratory, which may lead to less accurate estimates of seroprevalence compared with other tests. The lack of a gold standard reagent prevents us from definitively establishing the accuracy of different anti-*Ct* antibody tests; we currently rely on agreement between tests in population-based studies and triangulating those data with clinical and infection data to determine the best tests for use. The lack of infection data in these surveys therefore also limits our ability to interpret our data. Seropositivity to *Ct* antigens may result from transient exposure to urogenital strains, but these data revealed clear differences in seropositivity and SCR in the single district with TF > 5% compared with those < 5% TF. There is a certain degree of subjectivity in reading the LFA, so despite strong population-level agreement amongst the different tests, it is difficult to disentangle whether between-test variation is due to test performance or inter-rater disagreement.

In baseline mapping of two districts in Togo, population-level estimates for seroprevalence and SCR were essentially equivalent when using the Pgp3 MBA, CT694 MBA, and the Pgp3 black latex LFA. Estimates were 2–3 times higher using the Pgp3-gold dipstick LFA or the LFA-cassette in Keran. The LFA-cassette had previously shown poorer sensitivity to detect antibodies in individuals known to be ocular-Ct-positive at the time of blood collection^15^ and incorporation of data based on use of this test with whole blood resulted in poor model fits in latent class analysis^31^. We have therefore stopped using this test in the field; it is unlikely to be necessary to have point-of-care testing for trachoma surveillance as programmatic decisions are made at the EU, rather than individual, level^15^. The Pgp3-gold dipstick LFA differed only slightly from the MBA and the prevalence estimates generated using it had overlapping 95% CIs with those generated using the black latex LFA (the latter having been developed in response to population-level serosurveys in which presumed false positives and poor inter-rater agreement were problematic). Additional comparison with the Pgp3 MBA in 2 health zones of DRC again show strong agreement between MBA-derived seroprevalence, SCR and Pgp3 black latex-derived data. These data, coupled with other as-yet-unpublished data, provide support for using black latex as a developing reagent for the Pgp3 LFA. Each test has advantages: MBA can be used for integrated serosurveillance and provides robust semi-quantitative data, whereas the LFA does not require advanced instrumentation and can be run in essentially any laboratory. Having tests available on multiple platforms will allow national trachoma programs flexibility in selecting the best one for their particular need, and we have presented here further improvements to the test arsenal for future use in population-level serosurveys.
