# Publisher Correction: Comparison of platforms for testing antibodies to *Chlamydia trachomatis* antigens in the Democratic Republic of the Congo and Togo

**DOI:** 10.1038/s41598-021-97582-z

**Published:** 2021-09-01

**Authors:** Sarah Gwyn, Marcel S. Awoussi, Ana Bakhtiari, Rachel N. Bronzan, Kathryn Crowley, Emma M. Harding-Esch, Yao Kassankogno, Janvier N. Kilangalanga, Felix Makangila, Sylvain Mupoyi, Jeremiah Ngondi, Bonaventure Ngoyi, Stephanie Palmer, Jessica M. Randall, Anders Seim, Anthony W. Solomon, Raymond Stewart, Kwamy Togbey, Pitchouna A. Uvon, Diana L. Martin

**Affiliations:** 1 Division of Parasitic Diseases and Malaria, Centers for Disease Control and Prevention, 1600 Clifton Rd NE B23 Room 10-113, Atlanta, GA 30029 USA; 2 Ministère de la Santé et de L’Hygiène Publique, Lomé, Togo; 3 Task Force for Global Health, Decatur, GA USA; 4 Health and Development International, Newburyport, MA USA; 5 RTI International, Washington, DC USA; 6 Clinical Research Department, London School of Hygiene & Tropical Medicine, London, UK; 7 Health and Development International, Lome, Togo; 8 Saint Joseph Hospital, Kinshasa, Democratic Republic of the Congo; 9 Ministry of Public Health, Kinshasa, Democratic Republic of the Congo; 10 RTI International, Kinshasa, Democratic Republic of the Congo; 11 FHI360, Washington, DC USA; 12 Emory University, Atlanta, GA USA; 13 Department of Control of Neglected Tropical Diseases, World Health Organization, Geneva, Switzerland; 14 Programme National des Maladies Tropicales Négligées, Ministère de la Santé et de l’Hygiène Publique, Lomé, Togo

Correction to: *Scientific Reports* 10.1038/s41598-021-86639-8, published online 31 March 2021

The Acknowledgements section in the original version of this Article was incomplete.

“The surveys in the Democratic Republic of the Congo were generously supported by the American People through the United States Agency for International Development (USAID) via its ENVISION project (cooperative agreement number OAA-A-11-00048) and Act to End NTDs | East program (cooperative agreement No. 7200AA18CA00040), implemented by RTI International. The surveys and activities in the Togolese Republic were generously supported by the American People through the USAID via its End Neglected Tropical Diseases in Africa project (cooperative agreement number AID-OAA-A-10-00050), managed by FHI 360. Laboratory work at CDC was funded through an interagency agreement with USAID.”

now reads:

“The surveys in the Democratic Republic of the Congo were generously supported by the American People through the United States Agency for International Development (USAID) via its ENVISION project (cooperative agreement number OAA-A-11-00048) and Act to End NTDs | East program (cooperative agreement No. 7200AA18CA00040), implemented by RTI International. The surveys and activities in the Togolese Republic were generously supported by the American People through the USAID via its End Neglected Tropical Diseases in Africa project (cooperative agreement number AID-OAA-A-10-00050), managed by FHI 360. Laboratory work at CDC was funded through an interagency agreement with USAID. **Disclaimer**: The authors alone are responsible for the views expressed in this article and they do not necessarily represent the views, decisions or policies of the institutions with which they are affiliated. The authors declare no competing interests.”

The original Article has been corrected.

